# Contrasting Life History Characteristics Between Riverine and Lacustrine Anadromous Arctic Char ( *Salvelinus alpinus* ) in the Western Canadian Arctic

**DOI:** 10.1002/ece3.72734

**Published:** 2025-12-19

**Authors:** Colin P. Gallagher, Xinhua Zhu, Ellen V. Lea, Kimberly L. Howland

**Affiliations:** ^1^ Fisheries and Oceans Canada Winnipeg Manitoba Canada; ^2^ Fisheries and Oceans Canada Inuvik Northwest Territories Canada

**Keywords:** growth, migration, mortality, size, trade‐offs, vital rates

## Abstract

Freshwater habitat characteristics are known to affect life history traits of migratory salmonids. Although the life cycle of the anadromous form of Arctic char ( *Salvelinus alpinus* ) is typically associated with lakes, there is a small number of anadromous populations in North America that spawn, rear, and overwinter exclusively in rivers. The life history traits of these relatively understudied populations and how they differ from lacustrine Arctic char are poorly documented. We characterized life history tradeoffs expressed by anadromous Arctic char originating from a riverine (Hornaday River) and a relatively nearby lacustrine system (Tatik Lake of the Kuujjua River) in the western Canadian Arctic using a 10‐year dataset. The riverine population attained smaller average size (600 mm vs. 628 mm, fork length) and mean age (7.7 vs. 10.6 years), had a lower longevity (14 vs. 26 years), and expressed a 44% higher growth rate resulting in larger size‐at‐age prior to reaching modeled length asymptote (700 vs. 754 mm). Furthermore, the riverine population had a younger modal age‐at‐maturity (approximately 6–7 vs. 11–13 years) and mean age‐at‐first migration (4.1 vs. 6.4 years), and a higher natural mortality rate (0.31 vs. 0.21 per year). Our results broaden knowledge on the spectrum of life history strategies exhibited by anadromous Arctic char and underscore how freshwater habitat influences vital rates and life history tradeoffs, which have implications for conservation and sustainable harvest (e.g., maximum sustainable yield) of salmonids.

## Graphical abstract

Freshwater habitat characteristics are known to affect life‐history traits of migratory salmonids. Comparison of a riverine and lacustrine population anadromous Arctic char revealed significant tradeoffs in traits such as size, age, growth, maturity, migration, and mortality. These results underscore how freshwater habitat influence vital rates and life‐history tradeoffs, which have implications for conservation and sustainable harvest of salmonids.

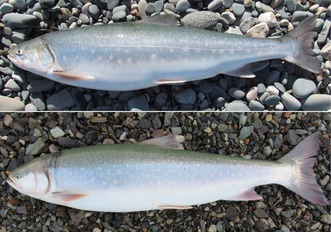

## 1 Introduction

Environmental conditions, including physical habitat characteristics, are known to influence the patterns of a species' life history such as population density, age, growth, reproduction, and survival (Roff 1992; Braendle et al. 2011). Aspects of life history are controlled by genetic and environmental factors, which operate within phylogenetic constraints such as developmental and physiological limits, resulting in trade‐offs for how an organism allocates limited energetic resources to maximize fitness (i.e., survival and reproduction) (Braendle et al. 2011; Oli and Coulson 2016). Comparisons of intraspecific life history strategies between riverine and lacustrine populations of fishes have revealed tradeoffs pertaining to size, size‐at‐maturity, and reproductive investment (Mazzoni and Iglesias‐Rios 2002; Giam and Olden 2018). Examining how variation in life history strategies can be influenced by habitats has implications for the conservation and management of both species and their habitats, particularly for fish species that exhibit a high degree of life history variation and complex spatio‐temporal patterns in habitat use (Wiedmann et al. 2014; Logez et al. 2016). This is particularly urgent for the Arctic where endemic fishes and their habitats are experiencing climate change at an unprecedented rate that is approximately three times faster than the global mean (Reist et al. 2006; Lam et al. 2016; Landrum and Holland 2020; Zhou et al. 2024).

Fishes from the Salmonidae family display a high degree of life history variation that can be expressed by ecological polymorphism, phenotypic plasticity, and alternative reproductive and migratory tactics (Hendry and Stearns 2004; Muir et al. 2013; Hutchings et al. 2019; Birnie‐Gauvin et al. 2021). Depending on life history strategies, salmonid species can occupy various habitats during their lifetime such as lakes, rivers, estuaries, and oceans, or a combination thereof (Arostegui and Quinn 2019). Arctic char ( *Salvelinus alpinus* ) is a cold‐adapted iteroparous salmonid with a Holarctic distribution that exhibits an exceptional amount of intraspecific variation in morphology, ecology, and life history (Klemetsen 2010, 2013). Arctic char inhabits freshwater systems although some populations residing in habitats connected to the sea are anadromous, performing seasonal migrations between freshwater spawning and overwintering habitats and highly productive marine feeding habitats in order to gain energetic and reproductive benefits (Johnson 1980). Its diversity in life history characteristics and tactics is a product of glacial cycles during the Pleistocene and the combination of an ability to occupy multiple ecological niches in post‐glacial habitats and tendency to disperse (e.g., migrate) to new locations (Moore et al. 2015; Weinstein et al. 2024). Arctic char inhabiting lakes have the propensity to exhibit polymorphic populations that have distinct growth and reproductive attributes to maximize fitness in alternative ecological niches and habitats (Sandlund et al. 1992; Smalås et al. 2013). Environmental conditions can have an important effect on Arctic char, where seasonal precipitation and temperatures influence growth (Chavarie et al. 2019; Svenning et al. 2024), lake primary production can affect the prevalence of anadromy in a population (Finstad and Hein 2012), and increased temperatures can decrease population abundance (Svenning et al. 2022). An additional example of environmental influences on life history properties is the latitudinal cline in growth (Chavarie et al. 2010) and fecundity (Power et al. 2005), both of which decline with increasing latitude, observed in anadromous populations of Arctic char in eastern North America.

Freshwater habitats and their characteristics (e.g., lacustrine and riverine) have an important effect on salmonid life history including growth, size‐at‐maturity, size‐ and age‐at‐first migration, and migration timing (O'Connell and Ash 1993; Dempson et al. 1996; Jonsson et al. 2001; Jonsson and Jonsson 2011a; Jensen et al. 2012; Pavlov et al. 2013). The Arctic char's life cycle is typically associated with lakes (DeLacy and Morton 1943; Johnson 1980; Dennert et al. 2016; Svenning et al. 2022), although the anadromous form uses rivers as a migration corridor between lacustrine and marine feeding habitats. Although relatively infrequent, anadromous populations that spawn, rear, and overwinter exclusively in a river have been found in multiple river systems in Europe (e.g., Nordeng 1983; Jensen 1994; Jensen and Rikardsen 2008; Siikavuopio et al. 2009; Svenning et al. 2012). In North America, riverine populations of Arctic char appear to be uncommon, with a small number of populations thus far documented in Northwest Territories (Harwood and Babaluk 2014), Nunavut (Moore 1975; Stewart 2005; Smith et al. 2022), Québec (Cunjak et al. 1986), and Labrador (Anderson 1985; Power and Dempson 2025). There is a paucity of research on the life history tradeoffs incurred by riverine Arctic char, particularly in North America. In Norway, a comparison between a lacustrine and riverine population revealed that riverine anadromous Arctic char had faster growth and a shorter lifespan, attained smaller sizes, and had a younger age‐at‐first migration (Jensen 1994). Examining the intraspecific diversity, including demographic rates, within anadromous Arctic char (e.g., Roux, Tallman, et al. 2011; Burke et al. 2022) is important to better account for and conserve the extent of the high variation expressed by the species (Klemetsen 2013; Reist et al. 2013).

**FIGURE 1:**
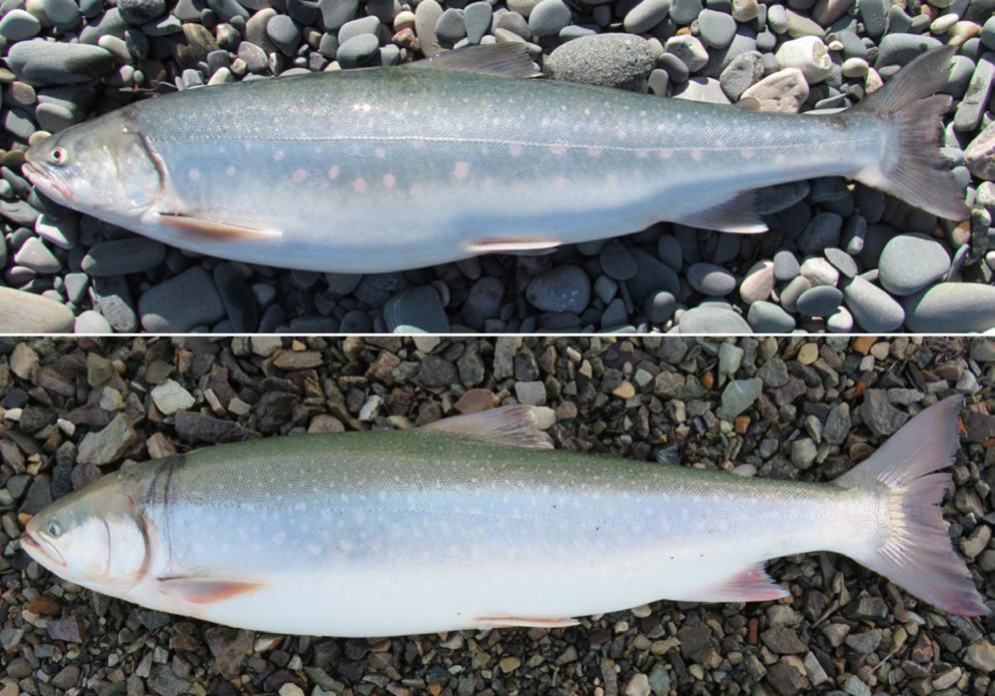
Anadromous Arctic char from Darnley Bay (riverine population) (550 mm fork length) (top) and Ulukhaktok area (lacustrine population) (> 600 mm fork length). Photo: Colin Gallagher.

We aim to compare life history properties of two relatively geographically proximate systems in the western Canadian Arctic with a long history of annual community‐based fisheries‐dependent monitoring programs where anadromous Arctic char (Figure 1) spawn, overwinter, and rear (pre‐smolt juveniles) in either a riverine or lacustrine habitat. We employ the terms ‘riverine’ and ‘lacustrine’ when referring to the anadromous life history from our two study systems for simplicity even though the terms are typically used in the context of describing non‐migratory life histories. Our objectives were to contrast demographic traits including size, growth, mortality, reproductive, and migratory characteristics. Even though riverine and lacustrine Arctic char both spend summers feeding and growing in different areas of the productive marine waters of the Amundsen Gulf, we predict the riverine population will exhibit a smaller size and younger age, faster growth rate, shorter lifespan, higher annual and natural mortality, and younger age‐at‐maturity and age‐at‐first migration. We expect our results to be consistent with the findings of Jensen (1994), who documented life history tradeoffs between riverine and lacustrine Arctic char in northern Norway (Beiarn and Saltdal rivers), whereby the riverine population exhibited a smaller size, younger age, faster growth rate, and a younger age‐at‐first migration. We hypothesize that contrasting life history properties between both study systems will be predominantly driven by freshwater habitat characteristics, which are known to influence key demographic traits (Jensen 1994; Jonsson and Jonsson 2011b). Rivers often have more variable conditions compared to lakes, such as fluctuating water levels, temperatures, and flow rates. Consequently, this will select for specific life histories of fish such as small‐bodied species with early maturation and low juvenile survivorship, which are associated with habitats with variable flow and adversely related to flow predictability and seasonality (Mims and Olden 2012). The paucity of riverine anadromous Arctic char populations in Arctic North America warrants the need to characterize their life history properties as a basis to understand how they differ from populations that originate from lakes and how such differences may affect their productivity and the fisheries they support. Life history traits such as growth rates, age‐at‐maturity, and migration impact a species’ vulnerability to fisheries, where slower growing species with longer lifespans and delayed maturation can be particularly susceptible to overfishing whereas fast‐growing species with shorter lifespans and higher reproductive output can withstand greater fishing pressure (Denney et al. 2002; Juan‐Jordá et al. 2015). Elucidating interrelationships between life history patterns and freshwater habitats is a salient topic for the conservation and sustainable harvest of salmonids (Jonsson and Jonsson 2011b; Roux, Tallman, et al. 2011), particularly for populations inhabiting dynamic riverine habitats where climate change is expected to have strong impacts on salmonid productivity (Gallagher et al. 2022).

## 2 Materials and Methods

### 2.1 Study Area

**FIGURE 2:**
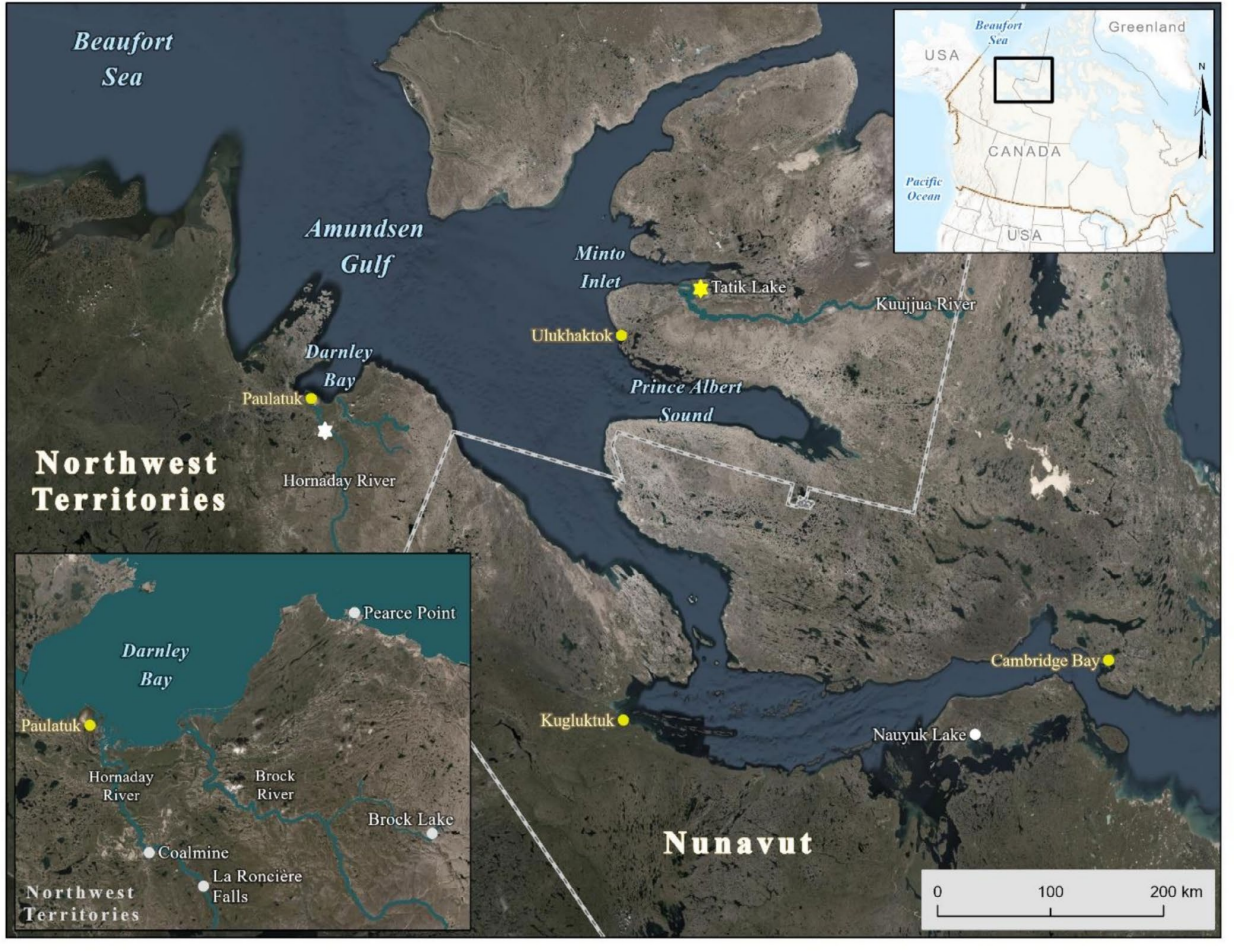
Study area in the western Canadian Arctic illustrating location of the Hornaday River (white star) and Kuujjua River (Tatik Lake) (yellow star), Northwest Territories. Lower left inset shows more details of Darnley Bay and the adjacent freshwater bodies (e.g., Hornaday River) while the upper right inset shows the location of the study area within Canada.

The Hornaday River, Northwest Territories, Canada, principally flows in the Tundra Plains ecoregion (Hornaday Upland Ecoregion) and occupies a wide valley with both glacial and river deposits (Ecosystem Classification Group 2012) (Figure 2). The lower reaches of the river are characterized by steep canyons with vertical walls rising up to 100 m (Zoltai et al. 1992) and La Roncière Falls, which is a high (23–38 m) impassable upstream barrier to fish located approximately 65 river km upstream from the mouth of the Hornaday. The width of the meandering river below the falls varies considerably and is generally between approximately 50 and 100 m and flows into Darnley Bay (Amundsen Gulf) 12 km from the coastal hamlet of Paulatuk (Figure 2). Arctic char from the Hornaday River enter the ocean in June (Harwood and Babaluk 2014) and remain to feed until approximately early to late August (Gallagher et al. 2017). Anadromous Arctic char have been documented to overwinter in a stretch of the Hornaday River with groundwater springs approximately 39 river km upstream of the mouth and also in a deep channel of the river's estuary/delta approximately 7 km from the ocean (Harwood and Babaluk 2014). Other species inhabiting the river include broad whitefish ( *Coregonus nasus* ), Arctic grayling ( *Thymallus arcticus* ), and longnose sucker ( *Catostomus catostomus* ) (Sutherland and Golke 1978).

The Kuujjua River, situated on Victoria Island, Northwest Territories, Canada, flows in a south‐west direction for over 350 km until draining into Minto Inlet (Amundsen Gulf) (Figure 2). The lower Kuujjua River consists of a broad valley surrounded by high bedrock cliffs and steep slopes with low‐growing vegetation (Ecosystem Classification Group 2013). Near its mouth, the Kuujjua River widens into a series of three separate lakes. Tatik Lake is the largest (42 km^2^) and furthest lake downstream (Figure 2). Although published bathymetric information for Tatik Lake is limited, depths generally range from approximately 5–15 m, although some areas are 25–30 m (H. Pettitt‐Wade, unpublished data). Anadromous Arctic char are known to spawn and overwinter in Tatik Lake, which is the most important location for winter subsistence fisheries for residents from the hamlet of Ulukhaktok (Gallagher, Howland, Papst, et al. 2021; Lea et al. 2023). Currently, there is no indication Arctic char move into the other lakes upstream of Tatik Lake in the Kuujjua River (Harwood et al. 2013; Hollins et al. 2022); however, anadromous Arctic char can also spawn in a small lake (locally known as ‘Red Belly Lake’) that is connected by a small stream that drains into western Tatik Lake, which requires the fish to swim approximately 47.3 river km. Altogether, some Arctic char from Tatik Lake, depending on their reproductive status, may experience a freshwater migration of up to 70.3 km. The distance between the outlet of Tatik Lake and the mouth of the Kuujjua River (i.e., river migration distance for anadromous Arctic char overwintering in the lake) is 16 river km. Acoustic telemetry data indicate Arctic char from the Kuujjua River enter the ocean in late June and remain in the marine environment until approximately mid‐August (Hollins et al. 2022). Other species inhabiting Tatik Lake include lake trout ( *Salvelinus namaycush* ) and lake whitefish ( *Coregonus clupeaformis* ).

The distance between the freshwater overwintering locations of the Hornaday and Kuujjua river stocks of anadromous Arctic char is approximately 340 km (Figure 1). The summer marine feeding locations for both populations are in the Amundsen Gulf of the eastern Beaufort Sea. Marine feeding habitats for both stocks are allopatric with no evidence of fish from one stock migrating to areas typically occupied by the other based on past external t‐bar tagging programs conducted in both populations during the 1990s to better understand spatial distribution and habitat use (Harwood and Babaluk 2014; L. Harwood, unpublished data). Specifically, the Hornaday River stock feeds in eastern and western Darnley Bay, and the Pearce Point area, which is immediately west of the entrance to the bay (Harwood and Babaluk 2014; McNicholl et al. 2020) (Figure 2). During summer, Kuujjua River Arctic char occupy locations that include Minto Inlet, the Ulukhaktok area, and western Prince Albert Sound (Hollins et al. 2022) (Figure 2).

The large embayments (Darnley Bay, Minto Inlet, and Prince Albert Sound) where Arctic char feed during summer are characterized as having similar biogeographic characteristics that are predominantly defined by the presence of landfast ice (which can cover 98% of the area and remain until the first half of July), an absence of multi‐year ice, and a freeze‐up that occurs in October (Hodgson et al. 2015). The embayments are characterized as having sheltered coastal physiography, mixed shallow depths (< 500 m), and are influenced by the input of rivers (Hodgson et al. 2015). Outside the embayments in the pelagic area of the Amundsen Gulf, the depths are mainly 200–500 m with a stratified water mass consisting of an Arctic surface mixed layer above waters of Pacific and Atlantic origin (Carmack and Macdonald 2002). Limited published data are available to compare food availability for Arctic char and overall productivity between Darnley Bay and Ulukhaktok (Minto Inlet and Prince Albert Sound) areas, which could have implications for explaining differences in certain life history parameters (e.g., growth) between both populations. Biomass and catch‐per‐unit‐effort of cod species (*Boreogadus* sp.) captured in a research trawl net in both areas were variable among years but overlapping in their ranges, with some indications of higher abundance in Minto Inlet than the Darnley Bay area (Niemi et al. 2020; Halliday et al. 2022; Herbig et al. 2023). Zooplankton biomass (g m^−2^) data from ecosystem surveys (BREA‐Marine Fishes Project, Canadian Beaufort Sea Marine Ecosystem Assessment) performed between 2012 and 2019 indicate that Darnley Bay, Minto Inlet, and Prince Albert Sound were primarily dominated by copepods (> 80%), pteropods (< 5%), amphipods (approximately 5%), and chaetognaths (approximately 5%), although more amphipods (approximately 8%) and chaetognaths (approximately 10%) were observed in a single year survey of Prince Albert Sound (A. Niemi, Fisheries and Oceans Canada, unpublished data). In addition, diversity (Shannon‐Weiner) of fishes and seafloor epifauna and infauna was observed to be similar among the three large embayments (A. Niemi, Fisheries and Oceans Canada, unpublished data).

### 2.2 Arctic Char Sampling

Life history data of Arctic char from the Hornaday River and Kuujjua River (Tatik Lake) populations were collected between 2010 and 2019 using annual community‐based fisheries‐dependent monitoring programs (see Harwood et al. 2013; Gallagher et al. 2017; Gallagher, Howland, Papst, et al. 2021). The research was conducted in accordance with all applicable laws and was authorized under Fisheries and Oceans Canada's (DFO) License to Fish for Scientific Purposes permits and supported by the Paulatuk and Olokhaktomiut Hunters and Trappers Committees. Both monitoring programs employed two Inuvialuit harvesters (termed ‘monitors’), selected by the local Hunters and Trappers Committee, who have expertise in the fisheries specific to their community. Most of the time for both programs, at least one person from each pair was consistently hired and performing the sampling among years. The monitors stationed themselves at a camp where fishing has traditionally occurred to primarily target Arctic char using single mesh gill nets (primarily 114 mm stretch mesh). The monitors were tasked with collecting catch‐effort and biological data not only from their own nets but also from others fishing in the same general area/waterbody. The monitors followed standardized sampling protocols and collected data on a daily basis over a relatively similar period of time among years. Both programs aimed to sample up to 200 Arctic char each year for fork length (±1 mm), round weight (±50 g), otoliths, sex, and current‐year reproductive status (i.e., whether the fish will spawn in fall or not (Hornaday River), or is currently spawning/post‐spawner (Tatik Lake)). Fisheries‐dependent monitoring at the mouth of the Hornaday River occurred between approximately late July and late August when migrating Arctic char enter the Hornaday River after feeding in Darnley Bay/Amundsen Gulf during spring and summer. Monitoring in Tatik Lake occurred mainly between October and November when harvesters set gill nets under the ice to harvest overwintering anadromous Arctic char.

The 10 years of data ensured an adequate sample size to statistically test for differences in life history properties between populations. To reduce bias, only the samples collected from 114 mm stretched mesh gill nets were used, which accounted for 78.2% and 90.4% of samples from Hornaday River and Tatik Lake, respectively.

### 2.3 Age Estimation

Ages were estimated using sagittal otoliths following the protocol outlined in Gallagher, Wastle, and Howland (2021) where otoliths were examined either whole (lateral side up) or thin‐sectioned (transverse plane). Otoliths of Arctic char from Tatik Lake were examined whole if ≤ age‐12 and thin‐sectioned if ≥ age‐13, while those from the Hornaday River were read whole if ≤ age‐9 and thin‐sectioned if ≥ age‐10 (Gallagher, Wastle, and Howland 2021). Otoliths were read whole by placing them in a small water‐filled glass petri dish over a black background and interpreted under a Leica MZ12.5 or a Nikon SMZ1000 dissecting microscope at magnifications of 10–80× with reflected light. Otoliths that met the criteria for thin‐sectioning were embedded with the sulcus side up in ColdCure Epoxy Resin (Industrial Formulators of Canada Ltd.). After hardening of the epoxy, the nucleus and section plane were delineated (see Gallagher, Wastle, Marentette, et al. 2021). A Buehler Isomet low‐speed saw (Lake Bluff, Illinois, U.S.A.) with two diamond wafering blades separated by a 0.5 mm spacer was used to acquire the section. Thin‐sectioned otoliths were examined using the same dissecting microscopes and light sources employed for whole otoliths. Annuli were identified based on criteria described by Chilton and Beamish (1982), where each annulus, representing one year of growth, consisted of an opaque (summer growth) and a translucent (winter growth) region. Otolith preparation and age estimation were performed in the same laboratory using the same pair of highly experienced age readers to either conduct primary reads or perform quality control where approximately 15% of otoliths were randomly selected and read with the aim of a relative percent difference < 5% between readers for the total annual sample collected from each stock (Gallagher, Wastle, and Howland 2021).

### 2.4 Growth, Mortality, and Reproduction

The length‐weight relationship was estimated using:

$$W=a\;L^{b}$$

where *W* is the body weight in grams (g), *L* is fork length (mm), *a* is a constant, and *b* is a growth exponent. Two expressions of the von Bertalanffy growth function (VBGF) were used to model length‐at‐age:

$$L_{t}=L_{\infty }\left(1-e^{-K\left(t-t_{0}\right)}\right)\;\text{and}\;L_{t}=L_{0}+\left(L_{\infty }-L_{0}\right)\left(1-e^{-\mathrm{Kt}}\right)$$

where $L_{t}$ is the fork length at age t, $L_{\infty }$ is the asymptotic average length, *L*_0_ is the length at age 0, *K* is the growth rate at which the fish approaches the asymptotic size (i.e., growth coefficient), and $t_{0}$ is the theoretical age when a fish has a length of zero. The first equation was employed to estimate growth parameters while the second was used in estimates of natural mortality (see below). Growth was estimated in a Bayesian framework using Markov chain Monte Carlo simulation (MCMC) to overcome the low number of samples > age‐15 and < age‐5 obtained using the single mesh gillnets. The Bayesian model was fit using the BayesGrowth package (v. 0.3.6) (Smart and Grammer 2021) using R statistical platform (v. 4.2.2, R Core Team 2022). Four MCMC chains with 65,000 iterations and a burn‐in period of 2500 iterations were run to explore the parameter posterior distributions. A thinning rate of 25 was applied to overcome possible autocorrelation between iterations. Model convergence was assessed by computing the Gelman‐Rubin statistic Rhat (i.e., ratio of the estimated variance between chains to the estimated variance within chains where values close to 1 indicates chains have converged while values > 1 indicates chains have not converged) and assessment of the mixing of the four MCMC chains were examined with diagnostic plots generated using a Bayesplot package (v.1.10.0) in R.

The growth of Arctic char from the Hornaday River and Kuujjua River (Tatik Lake) were modeled using Bayesian statistics, which applied a combination of informative and non‐informative prior distributions for the $L_{0}$ and $L_{\infty }$. Because no sources of information were available for populations in the Canadian Arctic, we used published size‐at‐hatching data from other systems to inform the $L_{0}$ prior. Pavlov and Osinov (2008) reported the average length of hatched Arctic char from Lake Davatchan, Russia, ranged between 15.0 and 16.5 mm while Beck et al. (2022) documented an average length ranging between 14.3 and 16.7 mm among Arctic char morphotypes in Iceland. To inform the prior for $L_{\infty }$, we used the maximum fork length of Arctic char from the Kuujjua River (880 mm) and the Hornaday River (835 mm) based on the archive of fisheries‐dependent data collected for both stocks. Furthermore information for the $L_{\infty }$ prior used information presented by Harris et al. (2021) who reported values of 754–1080 mm and 693–832 mm for anadromous Arctic in Halokvik and Jayko Rivers, respectively, which are located near the hamlet of Cambridge Bay (Ikaluktutiak), Nunavut (Figure 2). Given these information sources, the normally distributed priors (*μ*, *σ*) were set at $L_{\infty }$ ~ *N*(1000, 100) and *L*_0_ ~ *N*(16, 2.5). A non‐informative prior was used for process error *ɛ* and growth efficient *K*. The upper bound selected for the uniform distributions of *ɛ* and *K* was 100 and 0.5 year^−1^, respectively.

The Robson‐Chapman method was employed to estimate annual mortality (A) for both populations using the total combined catch‐at‐age data (2010–2019) on the descending limb of the catch‐curve (Chapman and Robson 1960; Ogle 2016). While estimates of natural mortality (*M*) (year^−1^) are important in the characterization of population dynamics, the difficulty in directly measuring the parameter requires using empirical estimators. The natural mortality tool (NMT, http://barefootecologist.com.au/shiny_m.html), a Shiny package for R, allows the application of 23 different empirical estimators of *M* based on 16 possible inputs of life history parameters (Cope and Hamel 2022). The data from both fisheries monitoring programs were used to estimate natural mortality for both populations (Table 2) (Gallagher et al. 2017; Gallagher, Howland, Papst, et al. 2021). Based on the VBGF parameters, the theoretical longevity (*T*_max_, years) can be estimated by:

$$T_{\max}=\left(\frac{1}{K}\right)⨯\ln\left[\frac{L_{\infty }-L_{0}}{\left(1-x\right)\times L_{\infty }}\right]$$

where *L*_∞_, *L*_0_, and *K* are VBGF parameters, and *x* is the proportion of *L*_∞_ reached at *T*_max_ for each age at which 95% and 99% of *L*_∞_ are reached (Dureuil et al. 2021).

Two of the 16 estimators for *M* in the Natural Mortality Tool utilize mean annual water temperature inhabited by a stock, which may indirectly influence *M* (Pauly 1980; Cope and Hamel 2022). To estimate a proxy for temperatures experienced by pre‐smolt Arctic char, we employed archived air temperature data given that stream temperatures in summer are highly dependent on solar radiation and air temperature (Saros et al. 2023). Air temperature data obtained from the Government of Canada's National Weather Network (https://climate.weather.gc.ca/historical_data/search_historic_data_e.html) for Paulatuk (Climate ID 2203058) and Ulukhaktok (Climate ID 2502505) were used to provide a relative proxy for spring and summer temperatures in the Hornaday River and Kuujjua River, respectively. Mean (±SD) daily air temperature between June 1 and September 30, 2010–2019 associated with Hornaday River and Kuujjua River was 7.28°C ± 5.20°C (range = −5.41°C–23.7°C) and 5.80°C ± 4.95°C (range = −6.44°C–20.8°C), respectively.

Female and male reproductive characteristics for both stocks were obtained from published reports (Gallagher et al. 2017; Gallagher, Howland, Papst, et al. 2021) and unpublished historical data. The tabulated attributes were fecundity (Hornaday River only) and egg diameter of current year spawning fish, age‐at‐maturity, and sex ratio (note, sex information from Tatik Lake in 2011 and 2016 was extremely and uncharacteristically biased towards males possibly because of issues with data recording). These extreme outliers were omitted from the data for the sex ratio calculations given the uncertainty whether they were properly recorded by the monitors. Modal age‐at‐maturity of males and females was determined by plotting the age distribution of fish that were identified by the monitors as ‘mature’ (i.e., current year spawners) to use as an indicator of the age when a majority of fish from a population typically became sexually mature. It was not possible to determine age‐at‐50%‐maturity because the proportion of immature and adult fish among age classes was not known given the difficulty of visually differentiating between sexually immature and adult current‐year non‐spawning (i.e., resting) gonads. No fecundity data were available for Arctic char from Tatik Lake; however, egg diameter data taken from a small number of spawning individuals collected in 1999 were available and are included here for comparison (L. Harwood; unpublished).

### 2.5 Laser Ablation Inductively Coupled Plasma Mass Spectrometry

Thirty‐two Arctic char otoliths from the Hornaday River and 31 from Kuujjua River (Tatik Lake) that were collected between 2010 and 2019 were randomly selected for otolith strontium (Sr) analysis. However, the youngest and oldest samples from both populations were included to ensure the full range of ages were examined. It is noted that Gallagher, Howland, Papst, et al. (2021) reported the anadromous Arctic char in Tatik Lake had a maximum age of 29 years; however, subsequent Sr analysis of the otolith of the fish revealed that it had never gone to sea during its lifetime. Subsequently, all samples aged > 25 years (*n* = 5) from the system were analyzed for annual Sr concentration to confirm the oldest anadromous age (26 years) (note, these were not in addition to the *n* = 31).

The otoliths examined for Sr. analysis were thin‐sectioned, embedded into acrylic rings (epoxy), polished, cleaned, and photographed. Laser ablation inductively coupled plasma mass spectrometry (LA‐ICP‐MS) analysis (LUV 213 laser and Thermo Finnigan Element 2 ICP‐MS) of otoliths was conducted at the Department Geological Sciences at the University of Manitoba. The ablation path was selected to perpendicularly cross annuli from the edge of the dorsal lobe to the core region (primordium) and then to the outer edge of the ventral lobe of the otolith to obtain annual ^86^Sr and ^43^Ca patterns throughout the entire life of the fish. The beam width was 30 μm and moved at a speed of 2 μm s^−1^ with a repetition rate of 20 Hz. A NIST 610 glass standard was analyzed every hour with 4 to 5 samples analyzed per hour while Sr. was internally standardized against calcium for ablation yield (constant Ca; in pure aragonite 40.02 wt%) and quantified against a NIST 610 external standard reference. Iolite (v. 2.21; Paton et al. 2011) was used to convert Sr counts per second to parts per million (ppm) by correcting to the Ca and NIST 610 standards. Sr profiles were overlaid on photographs of ablated otoliths to visualize variation in concentrations among annuli. Sr range (i.e., the difference between maximum and minimum Sr) was plotted against maximum Sr among the annuli ≥ 1 year to identify annuli exhibiting occurrences of ocean migration (Gallagher et al. 2018).

### 2.6 Statistical Analyses

The fisheries data sets from Kuujjua River (Tatik Lake) and Hornaday River (2009–2019) were combined and the data was explored to examine for outliers and test for normality of the variables (Zuur et al. 2010). Analysis of variance (ANOVA) was used to test differences among years for both sites and was performed with the R‐based package Rstatix v0.7.2 (Kassambara 2020), which is a pipe‐friendly framework for one or two‐way repeated measures. The Generalized Eta‐Squared (GES) statistic was used to report the effect‐size measure with statistical significance, such as type II one‐ and two‐way ANOVA (Olejnik and Algina 2003; Lakens 2013). Cohen (1988) suggests grouping the effect‐size measures into small (GES ≤ 0.02), moderate (GES = 0.13), and large (GES ≥ 0.26) categories. A comparison of length‐weight relationships was performed using the FSA (Simple Fisheries Stock Assessment Methods) package in R (Ogle et al. 2023) where log_10_ transformed length and weight data were pooled from both populations and fitted to a linear model with the population as a factor. Annual mortality (A) was calculated using the FSA package in R (Ogle et al. 2023). A chi‐squared test was used to evaluate for significant differences between the number of males and females between sites. The age‐at‐first migration and a number of lifetime ocean migrations were statistically compared using an unpaired two‐sample Wilcoxon test after confirming data were not normally distributed (Shapiro–Wilk test) following logarithmic transformation. One‐way and two‐way ANOVAs and Wilcoxon tests were performed at the critical level *α* = 0.05 (Zar 2010).

## 3 Results

### 3.1 Length, Weight and Age

**FIGURE 3:**
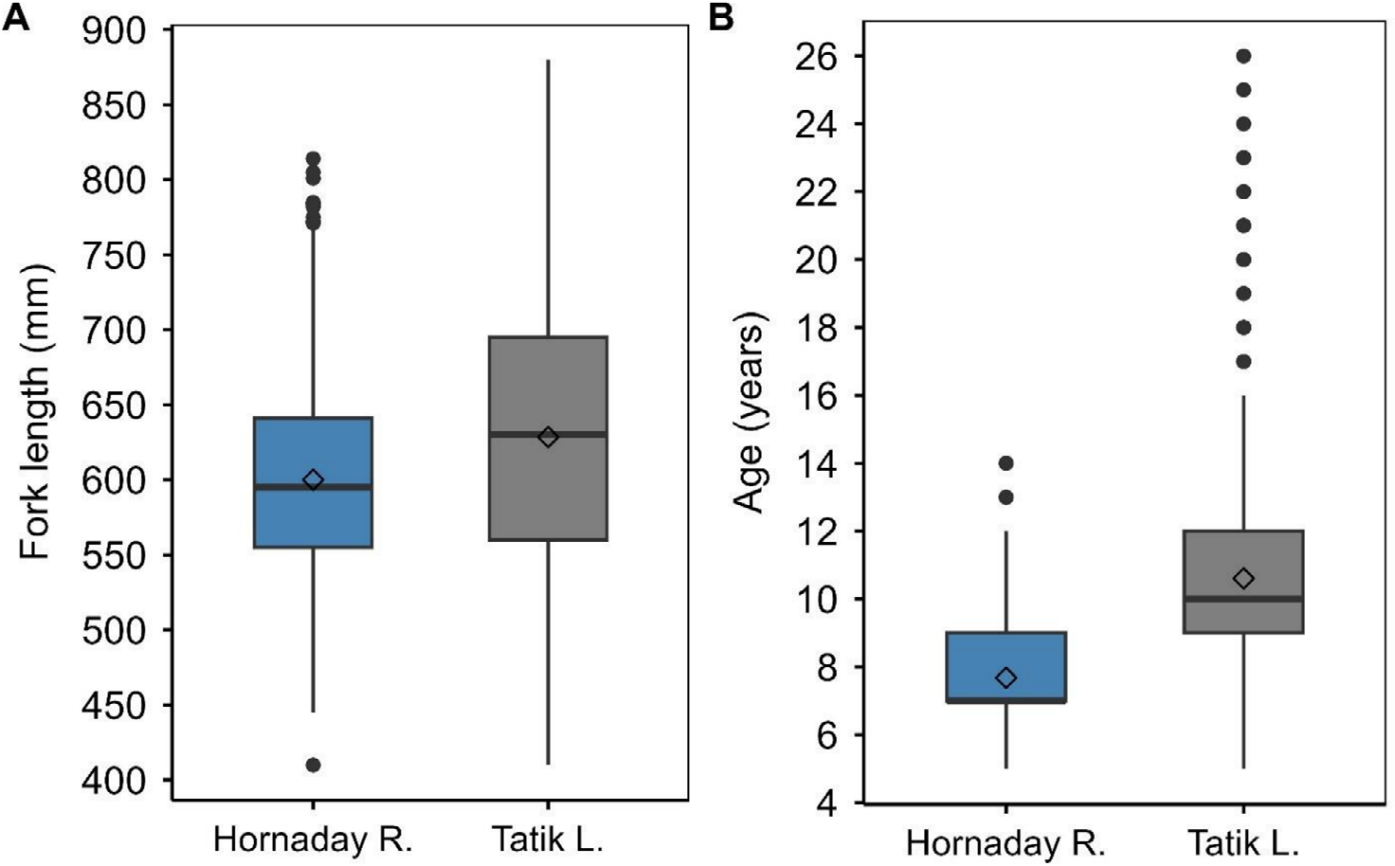
Box plot of (A) fork length and (B) age of anadromous Arctic char from the Hornaday River (riverine population) and Kuujjua River (Tatik Lake) (lacustrine population) captured in annual fisheries‐dependent monitoring programs between 2009 and 2019. Box plot illustrates median (−), mean (◊), quartiles (boxes), 1.5 × interquartile range (whiskers), and outliers (●).

Two‐way ANOVA indicated fork length varied among years with no clear pattern (*F*_3369,9_ = 21.81, *p* < 0.001, GES = 0.055) and location (*F*_3369,1_ = 89.89, *p* < 0.001, GES = 0.026), with a strong interaction between year and location (*F*_3369,9_ = 12.39, *p* < 0.001, GES = 0.032) (Figure S1). The average (±1SD) length of Arctic char sampled was higher in Tatik Lake: 622 ± 86 mm (range = 420–880 mm) compared to the Hornaday River: 598 ± 58 mm (range = 410–814 mm). Similarly, round weight varied among years with no clear pattern (*F*_3369,9_ = 17.20, *p* < 0.001, GES = 0.044) and location (*F*_3369,1_ = 100.19, *p* < 0.001, GES = 0.029), with a strong interaction between year and location (*F*_3369,9_ = 16.25, *p* < 0.001, GES = 0.042) (Figure S1). The average round weight (±1SD) was higher in Tatik Lake (3071 ± 1319 g; range = 700–7800 g) than in the Hornaday River (2697 ± 875 g; range = 850–6350 g). Age varied with year (*F*_3143,9_ = 27.86, *p* < 0.001, GES = 0.074) and location (*F*_3143,1_ = 1150.65, *p* < 0.001, GES = 0.268), with a strong interaction between year and location (*F*_3143,8_ = 10.39, *p* < 0.001, GES = 0.026). The average (±1SD) age was greater in Tatik Lake (10.4 ± 2.9 years; range = 5–24 years) compared to Hornaday River (7.6 ± 1.5 years; range = 5–14 years) (Table 1, Figures 3 and S2). The longevity of Arctic char between Hornaday River and Tatik Lake was markedly different (14 vs. 26 years) and similar outcomes were detected with the modeled longevity parameters (*T*_max_) (17.6 vs. 25.5 years) (Table 2).

**TABLE 1:** Summary statistics of fork length, round weight, and age of anadromous Arctic char from the Hornaday River (riverine population) and Kuujjua River (Tatik Lake) (lacustrine population) captured in annual fisheries‐dependent monitoring programs between 2009 and 2019.

	Fork length (mm)		Round weight (g)		Age (years)	
	Hornaday R.	Tatik L.	Hornaday R.	Tatik L.	Hornaday R.	Tatik L.
Mean (±1 SD)	600 (63.6)	628 (86.8)	2710 (982)	3173 (1344)	7.7 (1.5)	10.6 (3.0)
Median	595	630	2500	3000	7	10
Minimum	410	410	900	700	5	5
Maximum	814	880	7200	8000	14	26
*n*	1953	1525	1942	1513	1092	1525

**TABLE 2:** Life history parameters of anadromous Arctic char from the Hornaday River (riverine population) and Kuujjua River (Tatik Lake) (lacustrine population) captured in annual fisheries‐dependent monitoring programs between 2009 and 2019.

Life history parameter	Metric	Hornaday R.	Tatik L.
Length‐weight coefficient	a	3.33 × 10^−05^	8.90 × 10^−06^
	b	2.84	3.05
Longevity (observed)	Maximum age (years)	14	26
Longevity (modeled)	*T*_max_ (years)	17.62	25.47
Asymptotic length	*L*_∞_ ± SD (mm)	700.9 ± 6.7	754.1 ± 6.6
Growth rate	K ± SD	0.26 ± 0.01	0.18 ± 0.01
Age when *L* = 0	*t*_0_ ± SD	−0.12 ± 0.01	−0.09 ± 0.01
Mortality (per year)	Annual (±SE)	0.53 ± 1.57	0.32 ± 1.02
	Natural (±SD)	0.31 ± 0.02	0.21 ± 0.02
Reproductive	Fecundity (no. eggs; range)	1700–3300	No data
	Mean egg diameter (±SD) (mm)	3.4 ± 0.34	3.8 ± 0.36
	Range of egg diameter (mm)	2.8–4.1	3.3–4.2
	Female modal age‐at‐maturity (years)	7 (youngest = 4)	13 (youngest = 10)
	Male modal age‐at‐maturity (years)	6 (youngest = 5)	11 (youngest = 6)
Sex ratio	Male:Female	1.01:1	1.01:1
Ocean migration	Mean age‐at‐first (±SD) (years)	4.1 ± 2.0	6.4 ± 2.3
	Range of age‐at‐first migration (years)	2–11	3–13
	Mean number of lifetime migrations (±SD) (years)	4.6 ± 1.3	5.5 ± 2.9
	Range of number of lifetime migrations (years)	3–7	1–13
	Skipped migration prevalence (%)	9	12.5
Freshwater migration distance	Minimum (river kilometers) Maximum (river kilometers)	7 65	17 70.3

### 3.2 Growth

Statistically significant differences were detected in the slope (*F* = 12.99, df = 1, *p* < 0.001) and intercept (*F* = 1.35, df = 1, *p* = 0.2) of the length‐weight relationships between populations. The length‐weight relationship for Arctic char in Tatik Lake was:

$$W=9.05\times 10^{-06}L^{3.0435}\;\left(r^{2}=0.90\right)$$

while for fish from Hornaday River the relationship was:

$$W=2.48\times 10^{-05}L^{2.8884}\;\left(r^{2}=0.80\right).$$

The VBGF parameters derived using BayesGrowth for Arctic char in Tatik Lake were:

$$L_{t}=755.19\;\left(1–e^{-0.18\left(t+0.1164\right)}\right)$$

while for fish from the Hornaday River the parameters were:

$$L_{t}=700.87\;\left(1–e^{-0.26\left(t+0.0901\right)}\right).$$

**FIGURE 4:**
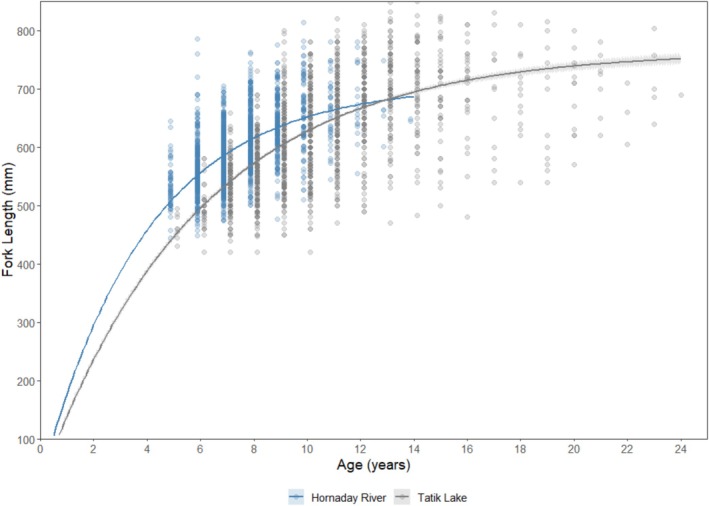
Length‐at‐age based on von Bertalanffy growth models of anadromous Arctic char from the Hornaday River (riverine population) and Kuujjua River (Tatik Lake) (lacustrine population) captured in annual fisheries‐dependent monitoring programs between 2009 and 2019.

There was a statistically significant difference in VBGF parameters of Arctic char between locations (Figure 4). Fish from the Hornaday system had 7% smaller asymptotic length (mean ± SD = 700.9 ± 6.6 mm) and 44% higher growth rate (mean ± SD = 0.26 ± 0.01 per year), compared to the asymptotic length (mean ± SD = 755.2 ± 6.7 mm) and growth rate (mean ± SD = 0.18 ± 0.01 per year) of the fish in Tatik Lake.

### 3.3 Annual and Natural Mortality

**FIGURE 5:**
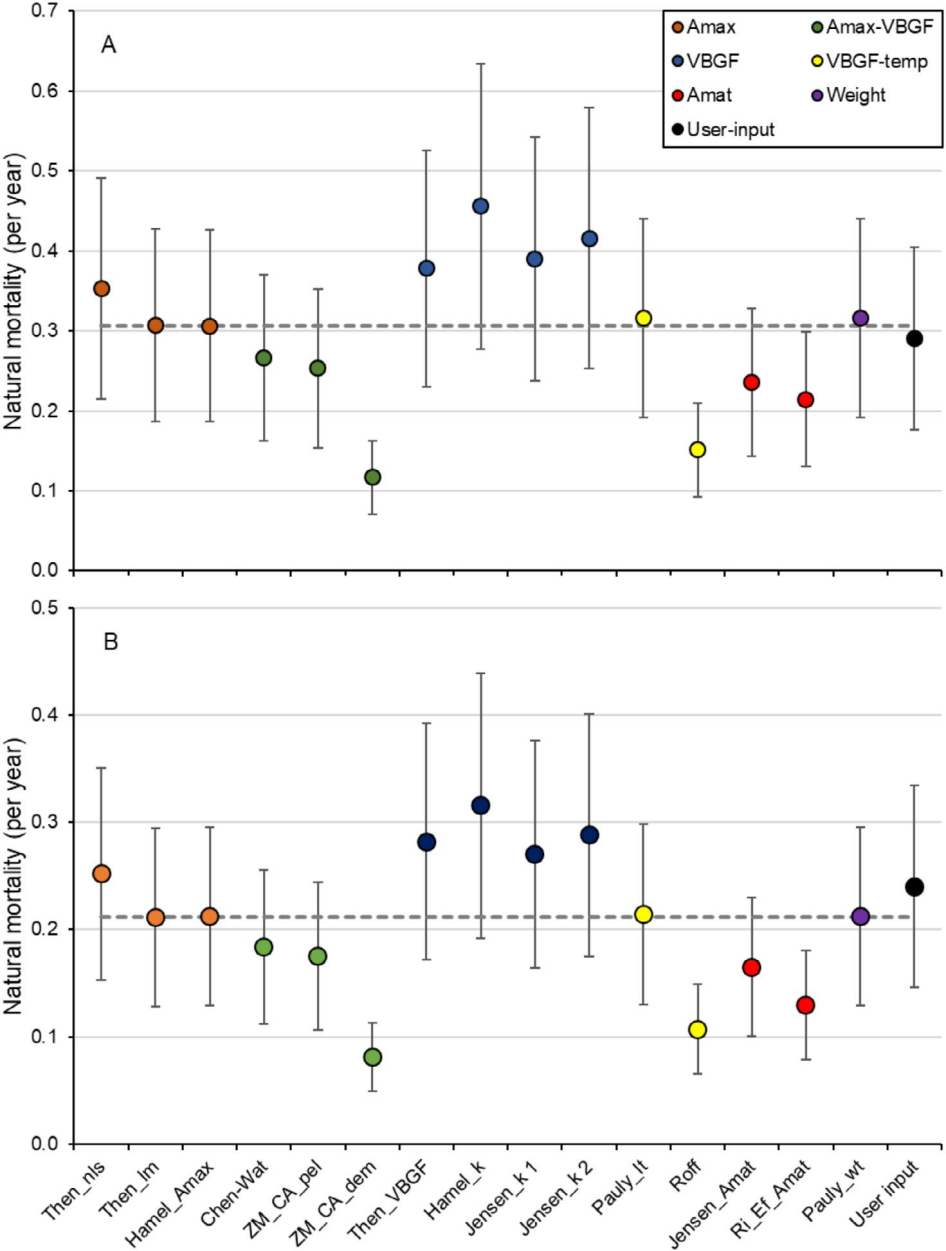
Point (with 95% normal error bars) estimates of natural mortality rate among 16 methods described by Cope and Hamel (2022) for Arctic char from (A) Hornaday River (riverine population) and (B) Kuujjua River (Tatik Lake) (lacustrine population). Colors in the point estimate panels refer to the respective groups of estimators. Orange: Maximum age; green: Maximum age and von Bertalanffy growth function; blue: Von Bertalanffy growth function; yellow: Von Bertalanffy growth function and water temperature; red: Age‐at‐maturity; purple: Body weight; and black: User input. Dashed line is the mean value among points estimates.

The annual mortality (±SE) of anadromous Arctic char from the Hornaday River was higher (0.53 ± 1.57) compared to Tatik Lake (0.32 ± 1.02) (Table 2). The natural mortality point estimates (±SE) varied among empirical methods (Figure 5). Based on the combination of sixteen methods, the analyses revealed the mean (±SD) natural mortality of Hornaday River Arctic char (*M* = 0.31 ± 0.02 per year) was 43% greater than in Tatik Lake (*M* = 0.21 ± 0.02 per year).

### 3.4 Reproduction and Sex Ratio

Although no fecundity data were available for Tatik Lake, current‐year spawning females from the Hornaday River ranged 1700 to 3300 eggs (Gallagher et al. 2017). Mean egg diameter was very similar between populations (approximately 3.6 mm) with a high degree of overlap in the range of values (Table 2). Females and males from the Hornaday River tended to mature at younger ages (approximately 7 and 6 years, respectively) compared to Tatik Lake (approximately 13 and 11 years, respectively) (Table 2). Anadromous males and females were found in nearly equal proportions in both populations (1.01:1). No statistically significant differences were observed in the frequency of males and females when comparing both populations (*X* ^2^ (d.f. = 1, *n* = 2182) = 0.0004, *p* = 0.98).

### 3.5 Ocean Migration

**FIGURE 6:**
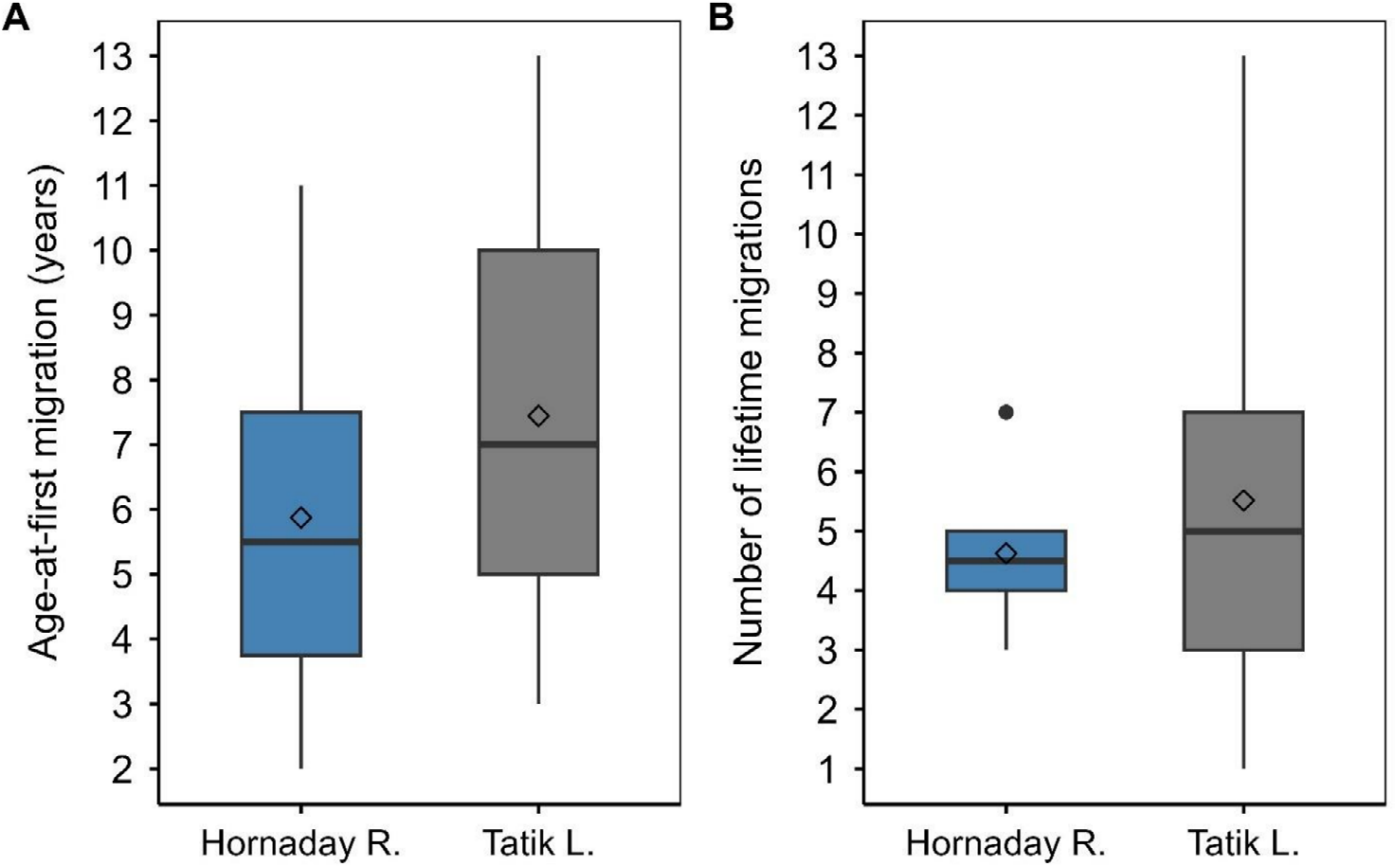
Boxplot of (A) age‐at‐first ocean migration and (B) lifetime number of ocean migrations of anadromous Arctic char from the Hornaday River (riverine population) and Kuujjua River (Tatik Lake) (lacustrine population). Box plot illustrates median (−), mean (◊), quartiles (boxes), 1.5 × interquartile range (whiskers), and outliers (●).

Both populations of anadromous Arctic char demonstrated differences in ocean migration characteristics (Table 2; Figures 6 and S3). The Hornaday River stock migrated for the first time at younger ages (mean ± SD = 4.1 ± 2.0) compared to Kuujjua (Tatik Lake) (mean ± SD = 6.4 ± 2.3) (Wilcoxon test; *W* = 819.5; *p* < 0.001). The oldest age‐at‐first migration was 13 years by a fish from Tatik Lake (Figure 6). The range of age‐at‐first migration overlapped considerably between both stocks (approximately age 3–11) even though it was uncommon for fish from the Hornaday >age‐5 to migrate for the first time (Figure 6). The total lifetime number of times at sea prior to harvest was similar between Arctic char from the Hornaday River (mean ± SD = 4.6 ± 1.3) and Tatik Lake (mean ± SD = 5.5 ± 2.9) (Wilcoxon test; *W* = 596; *p* = 0.16) even though the range in number of seaward migrations appeared quite different (Hornaday 3–7 years vs. Tatik 1–13 years) (Figure 6). The incidence of skipped ocean migration was low with only 9.4% (*n* = 3 fish; two skipping once and one skipping twice during lifetime) and 12.5% (*n* = 4 fish; each skipping once during lifetime) of Arctic char from the Hornaday River and Tatik Lake, respectively, exhibiting this behavior, with similar stock‐specific frequencies of individuals that skipped and did not skip migration during their lifetime (*X* ^2^ (d.f. = 1, *n* = 63) = 0.20, *p* = 0.96).

## 4 Discussion

The examples of western Canadian Arctic anadromous Arctic char stocks in this study that overwinter in riverine versus lacustrine freshwater habitats demonstrated distinct life history characteristics pertaining to size, growth, mortality, reproduction, and migration. The riverine stock attained smaller sizes and had shorter lifespans, faster growth, higher natural and annual mortality, younger age‐at‐maturity, and initiated ocean migrations at younger ages compared to the lacustrine population. Our study contributes to the documentation of life history plasticity exhibited by the anadromous form of Arctic char near the western edge of their geographic distribution in the Canadian Arctic, particularly for natural mortality, age‐at‐first migration, and lifetime frequency of ocean migrations, given the dearth of information on these parameters. Our results underscore how freshwater habitats can influence Arctic char vital rates and age‐at‐first migration, and the resulting life history tradeoffs. The tradeoffs inform the pace‐of‐life (i.e., fast‐slow life history continuum) (Healy et al. 2019) and life history variation within the anadromous form of Arctic char, which has received less attention compared to the intra‐specific life history diversity associated with non‐migratory polymorphic populations in lakes. Furthermore, our findings have implications on considerations relevant for the management of riverine Arctic char stocks and how population productivity may respond to climate change effects in freshwater habitats.

The life history differences between riverine and lacustrine life histories have important consequences for management of Arctic char fisheries. Riverine populations may be less productive (i.e., lower population abundance) compared to lacustrine ones (e.g., Dempson et al. 1996), particularly given the possibility that limited overwintering habitat in Arctic rivers may produce density‐dependent competition resulting in lower survival, as evidenced by the higher rate of natural mortality we observed. Indeed, population modeling indicates the maximum sustainable yield (MSY) of anadromous Arctic char from the Hornaday River is lower than Tatik Lake (2496 vs. 3543 fish) (DFO 2016; Zhu et al. 2017). While the higher growth rate, higher natural mortality, and lower estimated age‐at‐maturity exhibited by riverine Arctic char suggest these may be better able to withstand higher harvest rates based on a productivity‐susceptibility analysis of Arctic char stocks by Roux, Tallman, et al. (2011), the lower population abundance (depending on the size of freshwater system) of a riverine Arctic char population may reduce its ability to sustain prolonged periods of higher harvest rates compared to lacustrine ones (e.g., contrasting MSY). Other metrics such as lifetime reproduction events and spawning intervals, which are unknown in our two study populations, would also presumably differ between both freshwater habitat types and have an important effect on a population's ability to respond to harvest pressure (Juan‐Jordá et al. 2013).

Climate change effects on freshwater habitats will presumably have different consequences for riverine versus lacustrine populations of Arctic char. Arctic rivers are expected to be negatively affected by altered flow regime (e.g., timing of spring freshet), higher sedimentation rates, and increased temperatures (Box et al. 2019; Johnson et al. 2024). Under climate change, rivers exhibit an intensified hydrological cycle, amplifying seasonal (short‐term) differences in baseflow (Saros et al. 2023). In contrast, lakes are somewhat buffered against individual events but remain highly sensitive to cumulative, long‐term impacts from sustained warming and multiple stressors (Saros et al. 2025). Although speculative, the riverine life history of Arctic char may experience the effects of climate change in their freshwater habitats more acutely compared to the lacustrine life history, which could render these populations more vulnerable to conditions that disrupt critical processes such as spawning (e.g., optimal volume, substrates, and temperatures) and thereby impact aspects of their population dynamics (e.g., decreased egg and juvenile survival). Because climate‐related changes in Arctic freshwater carrying capacity for fishes will be exacerbated differently for riverine versus lacustrine habitats (e.g., Carey and Zimmerman 2014; Neuswanger et al. 2015), this will presumably have implications on population productivity of riverine/lacustrine Arctic char and the fisheries they support. Additional research is required to elucidate how climate change impacts to Arctic rivers and lakes could influence life history properties and persistence of salmonid populations (e.g., Leppi et al. 2023).

Our results demonstrating relative differences in size, age, growth, and migration between riverine and lacustrine populations of Arctic char were consistent with Jensen (1994) for anadromous Arctic char in northern Norway. The comparison by Jensen (1994) involved two populations that were geographically close to one another (the river mouths were 90 km apart), which revealed the riverine population had faster growth, smaller sizes, and younger ages, age‐at‐first migration, and maturation compared to the lacustrine stock. Although the population, environmental, and ecological characteristics of the systems between northern Norway and the western Canadian Arctic are considerably different, the similarities in the outcomes between Jensen (1994) and our study suggest these are due to the effect of variable freshwater habitat types in shaping the life history of anadromous Arctic char.

The life history characteristics of the Hornaday (riverine) stock show striking parallels with the closely related anadromous riverine char, Dolly Varden ( *S. malma* ), from similar latitudes in the western Canadian Arctic (Rat River population; mouth of Rat R. situated 67.76° N and drains into the Mackenzie Delta approximately 500 km southwest of Hornaday R. based on data collected from similar monitoring programs (Gallagher et al. 2020)). Specifically, Dolly Varden are relatively short‐lived (mean age = approximately 6 years; range = 3–13 years), experience high annual mortality (0.48), and undertake ocean migration at a young age (mainly 3–5 years of age) (Gallagher et al. 2018, 2020). Anadromous riverine char from the Coppermine River, which flows into the Coronation Gulf near the hamlet of Kugluktuk, Nunavut, approximately 390 km east from the mouth of the Hornaday River (Figure 2), is comprised of sympatric Arctic char and Dolly Varden (Reist and Sawatzky 2010; Weinstein 2023). Published life history data collected from fish captured using gill nets (various mesh sizes; data from 1981) demonstrated that mean age and longevity (mean = 8 years; range 3–15 years) (Gillman and Kristofferson 1984) were younger and more consistent with the Hornaday River (note, a low sample size of ages precluded robust estimation of annual mortality). Altogether, riverine chars in the western Canadian Arctic appear to demonstrate remarkably similar patterns in many key life history characteristics.

Among comparative lacustrine char stocks, the geographically proximate anadromous Arctic char stock from the Wellington Bay, Nunavut area (approximately 450 km southeast of Tatik Lake) near Cambridge Bay has a comprehensive life history data set. Harris et al. (2021) report Arctic char from the Halokvik River (sampled in the 2010s) attained large sizes ($L_{\infty }$ = 831 mm, *K* = 0.18) and old ages (mean = 14 years; range = 7–28 years), expressed a high age at maturity (approximately 10 years), and experienced relatively low annual (*A* = 0.30) and natural (*M* = 0.15; Zhu et al. 2021) mortality, consistent with the life history parameters of fish from Tatik Lake.

The observed tradeoffs in our study are consistent with what would be expected based on other studies looking at the interrelationships of life history traits in freshwater and marine species, with a smaller body size associated with higher mortality, faster growth, and earlier maturation (Forseth et al. 1995; Johnston and Post 2009; Wang et al. 2020). The higher natural mortality and lower longevity of the riverine Arctic char stock resulted in fish that grew faster, matured earlier, and undertook migration at earlier ages in order to maximize their fitness. The longer‐lived lacustrine population tended to start ocean migration at an older age and grew more slowly, which resulted in attaining larger sizes, which presumably would have conferred benefits of greater fecundity. When evaluating trade‐offs in the context of freshwater habitat as a template for life history (Jonsson and Jonsson 2011b), the differences observed between anadromous lacustrine and riverine Arctic populations of fishes of the genus *Salvelinus* suggest rearing, overwintering, and reproducing in relatively large lakes offer improved probability of survival and longevity. In contrast, fish inhabiting Arctic rivers contend with more seasonally variable and dynamic conditions such as flow rates (e.g., spring freshet), volumes (higher during summer open water vs. lower during winter ice‐covered), spring ice‐breakup, and water temperatures (Scrimgeour et al. 1994). In rivers like the Hornaday, spring ice‐breakup can produce moving ice jams and fronts with large pieces of fractured ice that can scour substrates and pose a risk of mortality to fish as they prepare to migrate. Riverine salmonids inhabit a more energetically demanding environment where competition may be high for potentially spatially limited spawning (adult) and overwintering (all life stages) habitats that do not freeze to the bottom during winter (Huusko et al. 2013). For example, the energetic content of Atlantic salmon ( *Salmo salar* ) parr rearing in lacustrine habitats was greater compared to those in riverine habitats, which was attributed to variable rearing conditions, contrasting prey densities, and differential energy expenditure that was assumed to have important implications for survival (Dempson et al. 2004). Salmonids in lacustrine environments, particularly for larger and deeper lakes such as Tatik, occupy more stable environments (e.g., habitats below the thermocline) where spawning locations are more predictable and overwintering habitat is less limited (Lennox et al. 2021).

Reproductive traits are a key feature in life history characteristics that are influenced by habitat, which are evident for Arctic char. Although the sample size was limited for robustly comparing egg diameter, we would have expected Arctic char from Tatik Lake to have larger mean egg diameter and greater fecundity given both are highly associated with increasing size in salmonids (Thorpe et al. 1984; Fleming and Gross 1990). Spawning frequency is unknown for either population although it is likely not performed annually given the high number (> 95%) of large‐bodied fish recorded as ‘immature’ (likely resting adults) in both stocks, which is consistent with other populations of Arctic char in Canada (Dutil 1986). Arctic char populations that display partial migration sometimes have a higher proportion of females among anadromous individuals given that costs and benefits of migration differ between sexes (Rikardsen et al. 1997; Hendry et al. 2004). Although speculative given the dearth of information regarding the extent to which Hornaday River and Kuujjua River (Tatik Lake) populations exhibit partial migration (i.e., limited data on resident non‐migratory fish), freshwater habitat type did not appear to influence the cost–benefit between the sexes given the sex ratio was equal between systems.

It is noted that the geographically nearest stock of anadromous Arctic char to the Hornaday River population is situated in the Brock River watershed (river mouths are < 20 km distance) (Roux, Harwood, et al. 2011) (Figure 2). The Arctic char in both systems differ genetically (Harris et al. 2016) yet both feed in Darnley Bay during summer. Inuvialuit harvesters from Paulatuk report that the Hornaday stock is considerably higher in population abundance compared to Brock River (Joe Illasiak, Paulatuk, Northwest Territories, pers. comm.). Fish in the Brock River are believed to overwinter in Brock Lake (area = 2.5 km^2^); therefore, it could have been ideal to use life history data of fish from this system (i.e., lacustrine overwintering habitat) to compare with the Hornaday River and remove the abovementioned issue of the potentially confounding effect of distance between study sites. Available Arctic char life history data from Brock Lake is limited to Roux, Harwood, et al. (2011) based on a small sample size (*n* = 19; most fish collected using gill nets with stretch mesh of 114 mm and 140 mm) where anadromy was confirmed for 16 fish using otolith strontium analysis that would have made a robust comparison challenging. The limited life history data from Brock Lake indicated that mean size, weight, and age was approximately 576 mm (maximum = 728 mm), 2246 g (maximum = 4400 g), 9.5 years (maximum = 14 years), respectively, and that age‐at‐first migration ranged 2–4 years (Roux, Harwood, et al. 2011). These characteristics are more similar to life history parameters of Arctic char from the Hornaday River than Tatik Lake. We posit that Brock Lake may not be an ideal lacustrine candidate system not only because of its small size but due to the lake's large littoral habitat (perimeter to area ratio = 6.6) (i.e., shallow) and small and spatially patchy profundal areas (≥ 20 m deep) accounting for 18% of total lake volume (Roux, Harwood, et al. 2011). It could be that the small size and low prevalence of deeper areas in the lake may render the habitat more riverine‐like resulting in anadromous Arctic char life history characteristics that are more similar to what was observed from the riverine (Hornaday) stock.

The age‐at‐first migration of Arctic char from Hornaday River and Tatik Lake overlapped with other populations in the Arctic. Studies of lacustrine Arctic char from the relatively nearby Coronation Gulf found mean age‐at‐first migration was age‐4 (range = 3–5 years) (Gilbert et al. 2016) and approximately age‐5 (range = 3–11) (Swanson et al. 2010) while the findings from populations in northern Nunavik, Canada revealed that migration began predominantly between age‐2 and ‐6 (range = 0–8 years) (Mainguy et al. 2023). High Arctic lacustrine populations at Svalbard, Norway and northern Ellesmere Island, Canada demonstrated a range of 4–13 years and 0–11 years, respectively (Radtke et al. 1996; Loewen 2016). The wide range of age‐at‐first and number of lifetime migrations in our study is consistent with the observation of a high degree of individual variation in migratory characteristics of northern iteroparous salmonids. This is likely driven by various factors including environmental characteristics affecting early‐life growth thresholds (Rikardsen and Elliott 2000; Morrison et al. 2021). Although the comparison between Hornaday River and Tatik Lake demonstrated statistically significant differences in age‐at‐first migration, the high degree of overlap for riverine Arctic char from the Hornaday River with abovementioned lacustrine populations underscores how the binary characterization of freshwater habitat type may not be as strong a predictor of first migration and that additional habitat‐related characteristics, including lake depth or river length, which is positively correlated with age‐at‐first migration for Arctic char in the Canadian high Arctic (Loewen 2016), likely influence migration behavior. While the tendency of the Hornaday River population to migrate for the first time at a younger age and presumably smaller size (we did not assess size‐at‐smoltification) might pose a greater risk of mortality from marine predators and stress from adjusting to the physiological stress of smoltling, this may be counteracted by spending more time in estuarine environments (Skilbrei et al. 1994; Handeland et al. 1996; Houde et al. 2019).

The infrequent instances of skipped migrations observed in our study were consistent with other northern populations (Radtke et al. 1996, 1997; Gilbert et al. 2016; Loewen 2016; Mainguy et al. 2023). Skipped ocean migrations of adult chars in the Arctic are associated with reproduction where a fish intending to spawn in the current year will forgo migration to the ocean and remain in freshwater during summer to spawn in fall (Johnson 1980; Gallagher et al. 2018). Arctic char from relatively nearby Nauyuk Lake, Nunavut (Figure 2), appear to exclusively exhibit a behavior of skipping ocean migration in the year they spawn (Johnson 1980; Dutil 1986). The low frequency of skipped migrations in our study populations suggests Arctic char exhibit a tactic of either skipping migration or migrating to the ocean in the same year they intend to spawn. Indeed, current‐year spawners sampled at the mouth of the Hornaday River when fish are leaving the marine environment typically account for < 5% of the annual sample (Gallagher et al. 2017), while Arctic char sampled in the marine waters of Ulukhaktok, which presumably consists of a high proportion of fish from Tatik Lake, can be comprised of approximately 23.6% current‐year spawners based on data calculated from Lea, Gallagher, et al. (2023). The interesting contrast between populations examined in our study, where Arctic char migrate from approximately 65 km to 70.3 km distances, and Nauyuk Lake, where Arctic char migrate a river distance of only 200 m to reach the sea, underscores the need for additional research into the interrelationships between migration and reproduction in adult Arctic char.

Comparing life history between a riverine and lacustrine population of anadromous Arctic char resulted in a contrast that revealed differences in pace‐of‐life attributes (i.e., a faster and slower type) that was consistent with results from studies of other species (e.g., smaller vs. larger body, earlier vs. later maturing, faster vs. slower growing). We proposed that freshwater habitat influenced these life history characteristics even though both populations feed in productive marine habitats in Amundsen Gulf during summer. While the pattern we observed was consistent with a similar study of Arctic char in Norway (Jensen 1994), research is needed to determine how riverine habitat characteristics such as watershed size and habitat volume, among many others, influence life history processes of Arctic salmonids that occupy rivers and lakes during their life cycle. As freshwater habitats in the Arctic are affected by climate change, it is imperative to consider how the life history characteristics of species will respond and the extent to which this will affect population productivity and long‐term viability, particularly for uncommon but important populations such as riverine Arctic char in North America.

## Author Contributions

**Colin P. Gallagher:** conceptualization (lead), data curation (lead), formal analysis (equal), funding acquisition (equal), methodology (equal), project administration (equal), visualization (equal), writing – original draft (lead), writing – review and editing (equal). **Xinhua Zhu:** formal analysis (equal), methodology (equal), visualization (equal), writing – original draft (supporting), writing – review and editing (equal). **Ellen V. Lea:** project administration (supporting), writing – original draft (supporting), writing – review and editing (equal). **Kimberly L. Howland:** funding acquisition (equal), project administration (equal), writing – original draft (supporting), writing – review and editing (equal).

## Funding

This work was supported by Fisheries Joint Management Committee. Fisheries and Oceans Canada.

## Conflicts of Interest

The authors declare no conflicts of interest.

## Supporting information


**Supplementary Figure 1.** Boxplots illustrating temporal variation in (A and B) fork length (mm) and (C and D) round weight (g) of anadromous Arctic char from the Hornaday River (A and C, riverine population) and Kuujjua River (Tatik Lake) (B and D, lacustrine population) captured in annual fisheries‐dependent monitoring programs between 2009 and 2019. Dashed blue line indicates the average value. Box plot illustrate median (−), quartiles (boxes), 1.5 × interquartile range (whiskers), and outliers (○).
**Supplementary Figure 2**. Frequency distribution of (A) fork length and (B) age of anadromous Arctic char from the Hornaday River (riverine population) and Kuujjua River (Tatik Lake) (lacustrine population) captured in annual fisheries‐dependent monitoring programs between 2009 and 2019.
**Supplementary Figure 3**. Frequency distribution of (A) age‐at‐first ocean migration and (B) lifetime number of ocean migrations of anadromous Arctic char from the Hornaday River (riverine population) and Kuujjua River (Tatik Lake) (lacustrine population).

## Data Availability

Biological data are uploaded as Supporting Information.
