# Supplementary material for: Contrasting Life History Characteristics Between Riverine and Lacustrine Anadromous Arctic Char ( Salvelinus alpinus ) in the Western Canadian Arctic
Source: Ecol Evol. 2025 Dec 19;15(12):e72734. doi: 10.1002/ece3.72734 (PMC12717139; doi:10.1002/ece3.72734)
Supplement: Supplementary file 1 — Supplementary Figure 1. Boxplots illustrating temporal variation in (A and B) fork length (mm) and (C and D) round weight (g) of anadromous Arctic char from the Hornaday River (A and C, riverine population) and Kuujjua River (Tatik Lake) (B and D, lacustrine population) captured in annual fisheries‐dependent monitoring programs between 2009 and 2019. Dashed blue line indicates the average value. Box plot illustrate median (−), quartiles (boxes), 1.5 × interquartile range (whiskers), and outliers (○). Supplementary Figure 2. Frequency distribution of (A) fork length and (B) age of anadromous Arctic char from the Hornaday River (riverine population) and Kuujjua River (Tatik Lake) (lacustrine population) captured in annual fisheries‐dependent monitoring programs between 2009 and 2019. Supplementary Figure 3. Frequency distribution of (A) age‐at‐first ocean migration and (B) lifetime number of ocean migrations of anadromous Arctic char from the Hornaday River (riverine population) and Kuujjua River (Tatik Lake) (lacustrine population). [file ECE3-15-e72734-s001.docx]

Supplementary figures


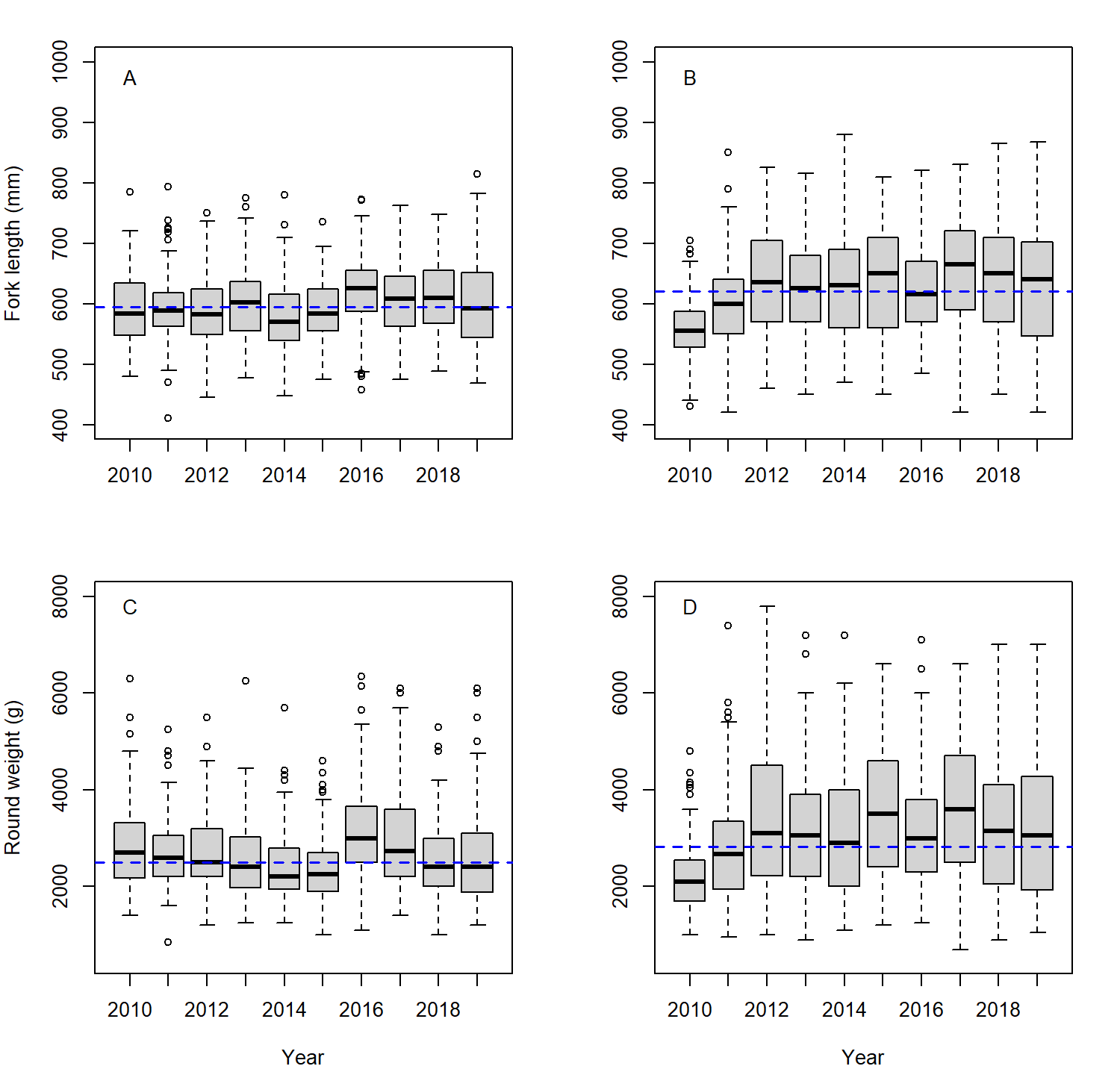


Supplementary Figure 1. Boxplots illustrating temporal variation in (A & B) fork length (mm) and (C & D) round weight (g) of anadromous Arctic char from the Hornaday River (A & C, riverine population) and Kuujjua River (Tatik Lake) (B & D, lacustrine population) captured in annual fisheries- dependent monitoring programs between 2009 and 2019. Dashed blue line indicates the average value. Box plot illustrate median (‒), quartiles (boxes), 1.5 x interquartile range (whiskers), and outliers (○).


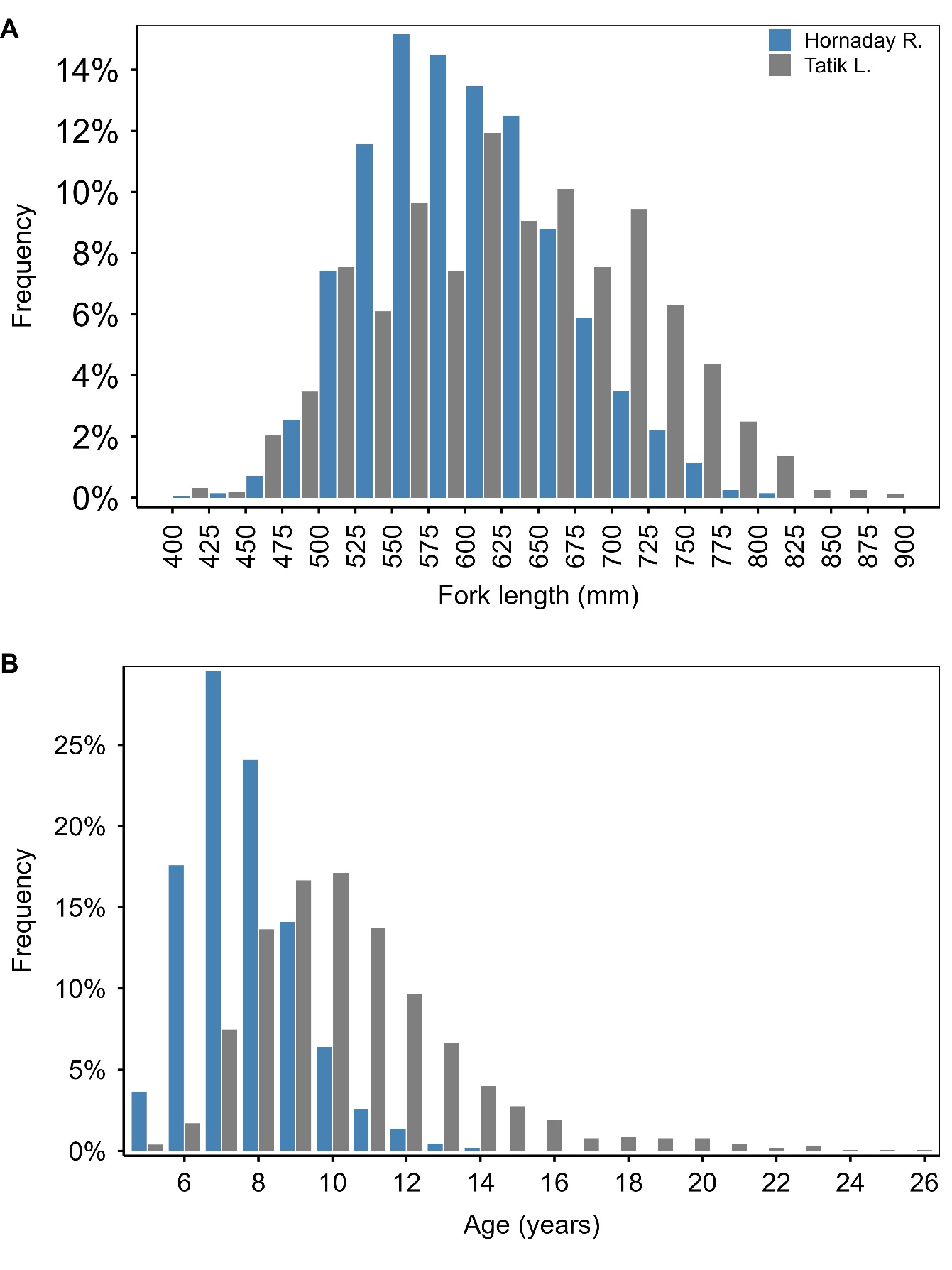


Supplementary Figure 2. Frequency distribution of (A) fork length and (B) age of anadromous Arctic char from the Hornaday River (riverine population) and Kuujjua River (Tatik Lake) (lacustrine population) captured in annual fisheries-dependent monitoring programs between 2009 and 2019.


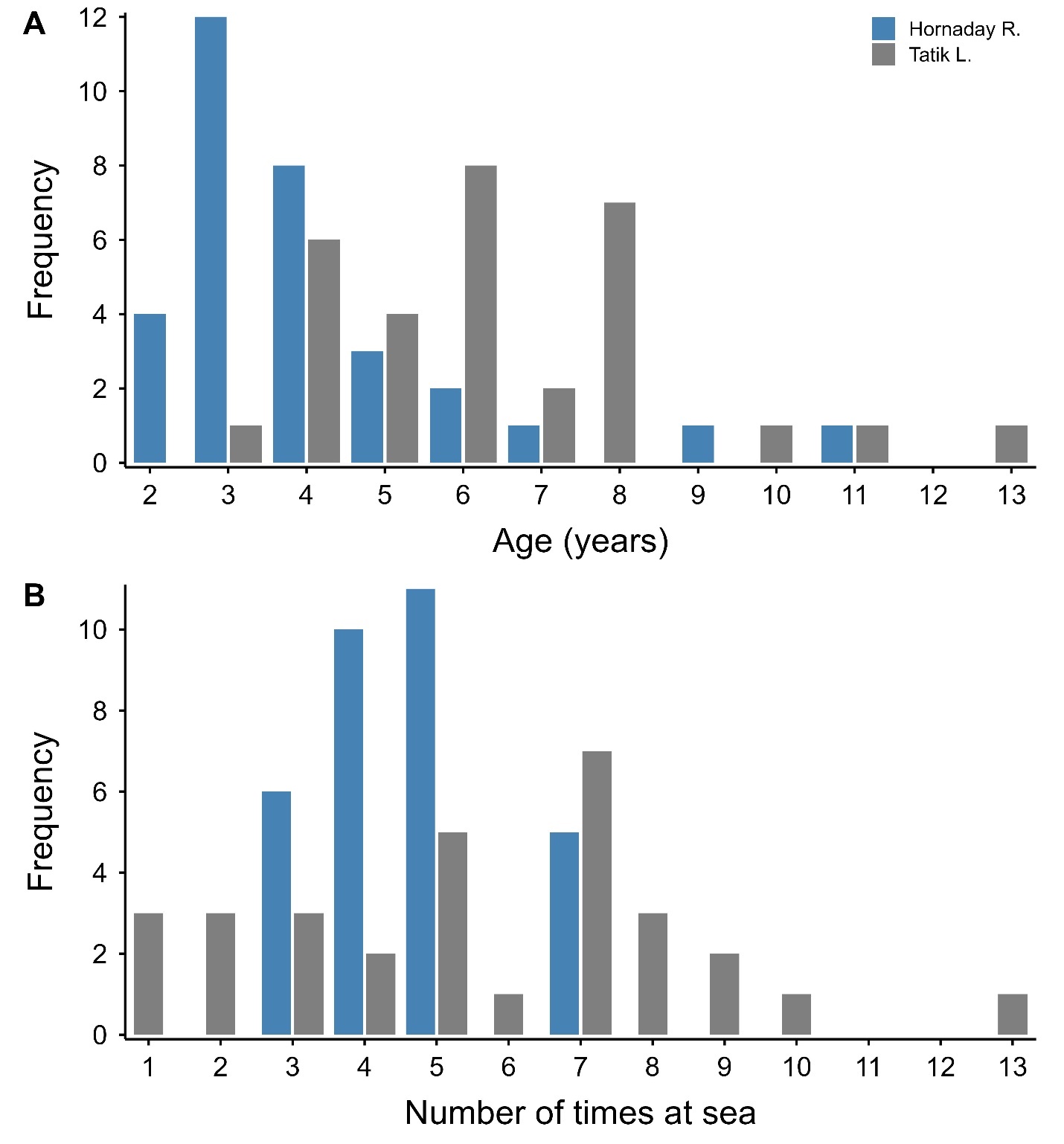


Supplementary Figure 3. Frequency distribution of A) age-at-first ocean migration and B) lifetime number of ocean migrations of anadromous Arctic char from the Hornaday River (riverine population) and Kuujjua River (Tatik Lake) (lacustrine population).
